# Investigating the associations between cognitive appraisals, emotion regulation and symptoms of posttraumatic stress disorder among Asian American and European American trauma survivors

**DOI:** 10.1038/s41598-022-22995-3

**Published:** 2022-10-28

**Authors:** Laura Jobson, Casey Willoughby, Philippa Specker, Joshua Wong, Adriana Draganidis, Winnie Lau, Belinda Liddell

**Affiliations:** 1 Turner Institute for Brain and Mental Health and School of Psychological Sciences, Monash University, Clayton, VIC 3800 Australia; 2 School of Psychology, University of New South Wales, Sydney, NSW 2052 Australia; 3 Phoenix Australia-Centre for Posttraumatic Mental Health and Department of Psychiatry, University of Melbourne, Carlton, VIC 3053 Australia

**Keywords:** Psychology, Human behaviour

## Abstract

This study investigated whether the associations between emotion regulation and cognitive appraisals and symptoms of posttraumatic stress disorder (PTSD) differ between Asian American and European American trauma survivors. Asian American (*n* = 103) and European American (*n* = 104) trauma survivors were recruited through mTurk and completed an on-line questionnaire assessing cognitive appraisals, emotion regulation and PTSD symptomatology. The European American group reported greater trauma-specific rumination, psychological inflexibility, seeking out others for comfort, and negative self-appraisals than the Asian American group. The Asian American group reported greater secondary control appraisals and cultural beliefs about adversity than the European American group. Second, cultural group moderated the associations between (a) brooding rumination, (b) fatalism, (c) self-blame, and (d) negative communal self-appraisals and PTSD symptoms. These associations were larger for the European American group than the Asian American group. Third, there was an indirect pathway from self-construal (independent and interdependent) to PTSD symptoms through certain emotion regulation approaches and cognitive appraisals. Additionally, cultural group was found to moderate several of these indirect effects. These findings highlight the importance of considering cultural background and cultural values in understanding the processes involved in PTSD. Further research in this area is needed.

## Introduction

Posttraumatic stress disorder (PTSD) is a disabling psychiatric disorder that has been observed in most societies and cultures^1^. The cognitive and emotional processes involved in the etiology, maintenance and treatment of PTSD have been researched at length, but while empirical advances have been impressive^2–4^, a significant limitation of this research is its predominant focus on Western cultural beliefs, norms and values^5^. This is problematic for several reasons. First, many trauma survivors do not identify with Western cultural values and in multicultural societies, such as the US, trauma survivors are from culturally diverse backgrounds. Second, research indicates that culturally tailoring mental health interventions improves treatment outcomes^6^. However, in the instance of PTSD there is very little evidence to guide this cultural tailoring. Third, cross-cultural research indicates that culture influences many of the psychological processes known to underpin PTSD^7,8^.

Two processes that have received considerable attention in the PTSD literature are emotion regulation and cognitive appraisals^2–4^. Emotion regulation is a broad construct^9^ that is complex, can be both habitual and situation-dependent, and includes emotion regulation abilities and strategies^10,11^. As the current study is one of the first studies to investigate culture, emotion regulation and cognitive appraisals in the context of PTSD, we adopted the definition of emotion regulation utilized in PTSD meta-analyses^4^; emotion regulation is the conscious or unconscious effort to affect the likelihood, duration or intensity of an emotion^9^. Additionally, we have focused on aspects of emotion regulation and cognitive appraisals that are prominent in the PTD literature. The PTSD literature highlights that PTSD symptoms are associated with significant difficulties in emotion regulation including an underutilization of adaptive emotion regulation strategies and an over-reliance on maladaptive strategies like emotion suppression, rumination, psychological inflexibility, and difficulties in emotion regulation^4,12,13^ (see Table 1 for definitions of the emotion regulation and cognitive appraisal variables considered in this study).

**Table 1:** Definitions of the emotion regulation and cognitive appraisals included in this study.

	Definition
**Emotion regulation**	
Emotion suppression	Inhibiting the outward expression of an emotion^19^
Emotional control values	Modulating one's own emotional experiences and expressions^58^
Psychological flexibility	Foundations traced to the tenants of Eastern philosophy and includes less rigid dominance of psychological reactions over chosen values and contingencies in guiding action^23,63^
Brooding rumination	Dwelling on the causes, consequences and meanings of negative emotions^19^
Interpersonal emotion Regulation	Using interpersonal relationships, or other people's presence while changing one's emotional state^62^
Difficulties in Emotion regulation	Difficulties in being able to regulate emotions^10^
**Appraisals**	
Primary control	Direct attempts to change one's current situation^64^
Secondary control	Attempts to change some aspect of the self and accept current circumstances^64^
Negative self	General negative view of the self^65^
Negative world	Negative appraisals about the world and mistrust of others^65^
Self-blame	Appraisals that the trauma happened because of the individual^65^
Fatalism	Acceptance of the situation and the belief that destinies are ruled by an unseen power^66,67^
Cultural beliefs about adversity	Beliefs which emphasize the positive value of adversity, people’s capacity to overcome adversity and people’s inability to change adversity and its negative impacts^33^
External appraisals	Challenges to beliefs and belonging^35^
Communal self	Dysfunctional appraisals about communal aspects of self and relationships^35^
Social/cultural self	Trauma leading to disintegration in one’s cultural/social roles^35^

PTSD is also associated with maladaptive cognitive appraisals, including cognitive appraisals of primary control, negative self-appraisals, negative appraisals about the world and self-blame^2,3,14,15^. It is important to note that cognitive appraisals can be considered an emotion regulation strategy (e.g., reappraising an experience can alter subjective emotional responses^4^). There are also reciprocal relationships between cognitive appraisals and emotion regulation; whereby deficits in emotion regulation can lead to stronger threat appraisals^16^ and maladaptive cognitive appraisals can disturb general emotion regulation processes^17^. Given the importance of cognitive appraisals in the development, maintenance and treatment of PTSD^2,4^ and following the approach of other PTSD researchers^17,18^, for the purpose of this study we have differentiated between emotion regulation and cognitive appraisals. Despite impressive advances in understanding the role of emotion regulation and cognitive appraisals in PTSD, researchers have predominately focused on trauma survivors from European American cultural backgrounds^7,19^. This is a concern because decades of non-clinical research demonstrates that cultural beliefs and values influence cognitive appraisals and emotion regulation^8,20^, including those highlighted to be of importance in PTSD.

Past non-trauma research indicates that Asian Americans and European Americans differ in emotion regulation and cognitive appraisals, which in turn influences the associations between these processes and psychological adjustment. Asian Americans report higher levels of rumination and suppression of emotion than European Americans^21,22^. Those with Asian heritage tend to have greater psychological flexibility^23^ and report higher use of interpersonal emotion regulation than those with European heritage^24^. Importantly, cultural group influences the association between emotion regulation and psychological adjustment. Rumination has a weaker association with psychological adjustment in Asian Americans when compared with European Americans^21^. Those from Asian cultural backgrounds, when compared to those from European backgrounds, are less likely to suffer poor psychosocial outcomes as a result of the suppression of emotion^25,26^. Engaging in interpersonal emotion regulation appears more beneficial for those with Asian cultural backgrounds when exposed to a stressful situation^24^. Turning to cognitive appraisals, Asian Americans report lower levels of perceived primary control than European Americans^27^ and perceived primary control is associated with less psychological distress for Asian American participants than European American participants^7,27,28^. Despite these findings, very little research has investigated the moderating role of cultural group (i.e., Asian American and European American) on the associations between both emotion regulation and cognitive appraisals and PTSD symptoms.

These cultural findings have been accounted for using theories of self-construal (i.e., how individuals perceive the self in relation to others^29^). Those with European cultural backgrounds tend to view the self as independent with a set of stable attributes and emphasizing agency^29^. In contrast, those from Asian cultural backgrounds tend to perceive the self as interdependent and emphasizing group harmony^29^. These differences in self-understanding shape an individual’s cognitions and strategies used to regulate emotions, which in turn influence psychological adjustment. Independent self-construal has been proposed to be associated with emphasizing individual experiences and expressing emotions^8,20^. Interdependent self-construal is associated with suppression and control of intense emotions, as these strategies are important for maintaining relatedness and group harmony^8,20,30^. Additionally, interdependent self-construal is considered to be associated with a more self-distanced approach to rumination, viewing the causes and consequences of one’s emotional states within a broader social context (rather than as simply relating to the self), while those valuing independence may engage in greater brooding related to the self and personal experiences^8,20,25,26,30,31^. This results in cultural differences wherein specific emotion regulation is deemed adaptive versus maladaptive in promoting mental well-being and reducing psychological distress^8,20^.

Self-construal has also been used to account for cultural differences in cognitive appraisals. Those valuing independent self-construal have been proposed to appraise experiences valuing personal control, independence and agency and such appraisal types are important for wellbeing^7,15,27–29,32^. In contrast, for those valuing interdependence there is less emphasis on these appraisal types and consequently such cognitive appraisals are less relevant to wellbeing ^7,29,32^. Instead, it has been proposed that those from Asian cultural backgrounds value cognitive appraisals of secondary control^32^, fatalism^7^, specific cultural beliefs about adversity^7,33^, psychological flexibility^23,34^ and public and communal self-appraisals^35^, as these cognitive appraisals relate to fitting in with, adapting to, and accepting the current situation, and thus, promote interdependent aspects of self-construal. Consequently, such cognitive appraisals may have greater influence on posttraumatic psychological adjustment among Asian Americans^7^.

Emerging research indicates that there are cultural differences in cognitive appraisals and emotion regulation in the context of PTSD. For European Australian trauma survivors, cognitive appraisals of personal primary control, negative self-appraisals, suppression and emotion dysregulation have been found to be associated with PTSD symptoms^19,36–39^. However, these cognitive appraisals and emotion regulation approaches have been found to be less relevant for the posttraumatic psychological adjustment of those from Asian cultural backgrounds^19,36–39^. Despite these findings, important questions remain regarding what emotion regulation and cognitive appraisals are of importance for trauma survivors with PTSD symptoms from Asian cultural backgrounds. While an initial study supported the notion that fatalism, cultural beliefs about adversity, and interpersonal emotion regulation may be valued in trauma survivors from Malaysia, and associated with PTSD symptom severity^38^, greater research is still needed to understand the relevance of these associations for Asian American groups.

This study aimed, therefore, to investigate whether the associations between emotion regulation and cognitive appraisals and PTSD symptoms differed between Asian American and European American trauma survivors. Specifically, we first aimed to investigate cultural group differences in emotion regulation and cognitive appraisals. Guided by the non-trauma cultural literature, we hypothesized that the Asian American group, when compared to the European American group, would score significantly higher on cognitive appraisals of secondary control, fatalism, cultural beliefs about adversity and public and communal self-appraisals^7,32,33,35^ and significantly higher on the emotion regulation approaches of emotional control, emotion suppression and interpersonal emotion regulation^20,25,26^. In contrast, we hypothesized that the European American group would report greater cognitive appraisals of primary control and negative self-focused appraisals^7,27,28^ and higher levels of the emotion regulation approach of psychological inflexibility^21,23^ than the Asian American group (“Hypothesis 1”).

**Figure 1:**
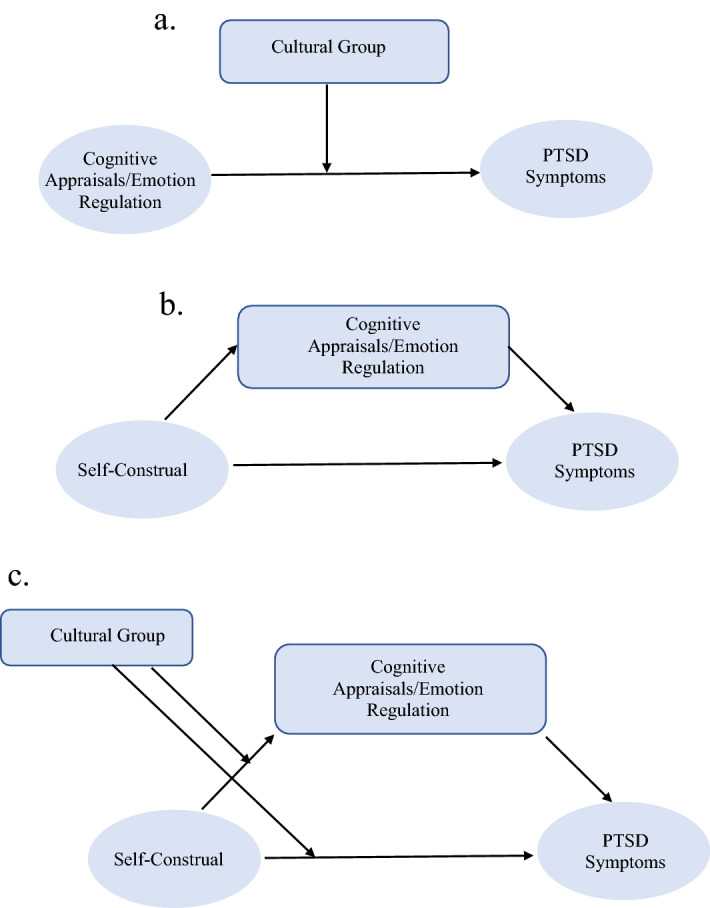
Figure depicting the moderation analyses (**a**), mediation analyses (**b**) and moderated mediation analyses (**c**).

Second, we investigated whether cultural group moderated the associations between (a) emotion regulation and (b) cognitive appraisals and PTSD symptoms (Fig. 1a). Again based on past non-trauma cultural research, we predicted the associations between certain cognitive appraisals (secondary control, fatalism, cultural beliefs about adversity, public and communal self-appraisals) and emotion regulation approaches (emotional control, suppression, interpersonal emotion regulation) and PTSD symptoms would be stronger for the Asian American group than the European American group. In contrast, we predicted the associations between certain cognitive appraisals (primary control, negative self-focused appraisals) and emotion regulation (brooding rumination, psychological inflexibility) and PTSD symptoms would be stronger for the European American group (“Hypothesis 2”).

Third, it has been highlighted that cross-cultural researchers should investigate both cultural group differences and cultural values^40^. Given self-construal is proposed to influence cognitive appraisals and emotion regulation, which in turn influences psychological adjustment^30,41^, we examined the indirect pathways between self-construal (independent and interdependent) and PTSD symptoms through emotion regulation and cognitive appraisals (Fig. 1b). We hypothesized that there would be an indirect association between independent self-construal and PTSD symptoms through cognitive appraisals and emotion regulation proposed to be associated with independent self-construal (e.g., psychological inflexibility, brooding rumination, primary control appraisals and negative self-focused appraisals). We also predicted there would be an indirect association between interdependent self-construal and PTSD symptoms through cognitive appraisals and emotion regulation that have been proposed to be associated with interdependent self-construal (e.g., suppression, emotional control, interpersonal emotion regulation, secondary control, fatalism, cultural beliefs about adversity and public and communal self-appraisals) (“Hypothesis 3”). While we recognize that cognitive appraisals and emotion regulation do not simply map onto independent versus interdependent self-construal, we have derived these hypotheses based on previous research and theoretical accounts of cultural differences reported in the non-trauma literature. We also explored whether cultural group moderated these indirect effects (moderated mediation, Fig. 1c).

We investigated these aims using a cross-sectional study. Those who had experienced a criterion A trauma and were residing in the US and identified as having European heritage or Asian heritage completed a battery of measures assessing emotion regulation, cognitive appraisals and PTSD symptomatology.

## Results

Sample characteristics are presented in Table 2. As shown in Table 2, the European American group was significantly older than the Asian American group. As expected, the European American group tended to score higher on independent self-construal than the Asian American group. However, unexpectedly, the two groups did not differ significantly on interdependent self-construal. The two cultural groups differed significantly in terms of education level but did not differ significantly in terms of gender distribution, index trauma type, time since trauma, or symptomatology. A significant proportion of each cultural group met clinical cut-off for probable PTSD diagnosis; 56 (53.8%) participants in the European American group and 53 (51.5%) participants in the Asian American group. Among the Asian American group, 78 participants (75.72%) were born in the US (second-generation migrants) and 25 participants (24.27%) reported being born in countries other than the US (including East Asia [China, Japan, South Korea] *n* = 12; South Asia [India, Pakistan, Bangladesh] *n* = 4, South East Asia [Philippines, Indonesia] *n* = 5, and North and South America [Canada, Guyana, Venezuela] *n* = 3) and had lived in the US for between 5 to 51 years (*M* = 29.10, *SD* = 11.24).

**Table 2:** Sample characteristics and group differences.

Variable	European American	Asian American	Findings
Age	40.83 (11.22)	37.04 (10.73)	*t*(205) = 2.48, *p* = 0.01
Gender Men:Women	57:47	56:46	χ^2^ (*N* = 207, *df* = 1) = 0.004, *p* = 0.95
Education^a^	24:17:54:8:1	13:4:58:24:2	χ^2^ (*N* = 207, *df* = 5) = 18.32, *p* < 0.01
Independent	4.92 (0.82)	4.74 (0.85)	*t*(205) = 1.55, *p* = 0.06 (one-tailed)
Interdependent	4.59 (0.87)	4.57 (0.91)	*t*(205) = 0.21, *p* = 0.42 (one-tailed)
Trauma type*	43:15:11:10:1:6:18	30:10:18:10:3:14:17	χ^2^ (*N* = 207, *df* = 6) = 9.89, *p* = .13
Time since trauma (years)	9.13 (1.13)	9.38 (1.07)	*t*(205) = 21.60, *p* = 0.11
PTSD symptoms	41.28 (20.49)	38.50 (17.16)	*t*(205) = 1.06, *p* = 0.29
Anxiety symptoms	8.27 (5.22)	7.48 (4.56)	*t*(205) = 1.16, *p* = 0.25
Depression Symptoms	6.62 (4.74)	5.58 (4.35)	*t*(205) = 1.65, *p* = 0.10
**Emotion regulation**			
Difficulties in emotion regulation	38.93 (15.29)	37.11 (13.50)	*F*(1,201) = 2.88, *p* = 0.09, η^2^ = 0.01
Emotional control	37.60 (6.99)	37.21 (5.610	*F*(1,201) = 0.28, *p* = 0.60, η^2^ = 0.001
Trauma-related rumination	26.68 (11.15)	22.33 (10.58)	*F*(1,201) = 9.63, *p* = 0.002, η^2^ = 0.05
Rumination (Brood)	13.11 (6.10)	12.77 (6.28)	*F*(1,201) = 1.72, *p* = 0.19, η^2^ = 0.01
Suppression	15.38 (5.52)	16.42 (5.12)	*F*(1,201) = 2.66, *p* = 0.10, η^2^ = 0.01
Psychological Inflexibility	24.94 (12.63)	22.24 (11.07)	*F*(1,201) = 6.02, *p* = 0.02, η^2^ = 0.03
IERQ			Wilks’ Lambda = 0.92, *F*(4, 198) = 4.62, *p* = 0.001, η^2^ = 0.09
Positive affect	17.37 (4.84)	16.33 (5.17)	*F*(1, 201) = 1.91, *p* = 0.17, η^2^ = 0.01
Perspective	12.76 (5.12)	12.45 (5.38)	*F*(1, 201) = 0.13, *p* = 0.72, η^2^ = 0.001
Soothing	15.36 (5.71)	12.81 (5.73)	*F*(1,201) = 12.22, *p* = 0.001, η^2^ = 0.06
Social modelling	15.37 (5.01)	15.24 (5.11)	*F*(1, 201) = 0.38, *p* = 0.54, η^2^ = 0.002
**Cognitive appraisals**			
Primary control	33.90 (13.94)	35.27 (12.87)	*F*(1, 201) = 0.47, *p* = 0.50, η^2^ = 0.002
Secondary control	51.67 (11.47)	53.76 (10.06)	*F*(1, 201) = 4.00, *p* = 0.047, η^2^ = 0.02
Pessimism	12.62 (3.89)	13.18 (3.56)	*F*(1, 201) = 3.66, *p* = 0.057, η^2^ = 0.02
Non-judgemental	5.50 (2.16)	5.14 (2.01)	*F*(1, 201) = 0.61, *p* = 0.44, η^2^ = 0.003
Cultural beliefs about adversity	33.14 (6.16)	35.31 (5.94)	*F*(1,201) = 10.14, *p* = 0.002, η^2^ = 0.05
Trauma related cognitions			Wilks’ Lambda = 0.89, *F*(3, 199) = 7.99, *p* < 0.001, η^2^ = 0.11
Negative self	59.08 (35.46)	51.26 (28.45)	*F*(1, 201) = 6.26, *p* = 0.01, η^2^ = 0.03
Negative world	26.19 (12.32)	29.46 (11.13)	*F*(1, 201) = 1.80, *p* = 0.18, η^2^ = 0.01
Self-blame	13.44 (8.76)	12.01 (6.84)	*F*(1, 201) = 3.15, *p* = 0.08, η^2^ = 0.02
Public and communal self			Wilks’ Lambda = 0.97, *F*(3, 199) = 2.38, *p* = 0.07, η^2^ = 0.04
External	19.11 (10.04)	18.17 (8.88)	*F*(1, 201) = 2.13, *p* = 0.15, η^2^ = 0.01
Communal	26.38 (12.10)	23.53 (11.23)	*F*(1, 201) = 6.28, *p* = 0.01, η^2^ = 0.03
Social/cultural	23.84 (11.19)	21.33 (8.88)	*F*(1, 201) = 4.80, *p* = 0.03, η^2^ = 0.02

*Accident: Natural Disaster: Non-sexual Assault: Sexual Assault: War/Kidnapping: Life-threatening Illness: Unexpected Death.

^a^Secondary: Post-Secondary: Undergraduate degree: Post-Graduate Degree: Prefer not to say.

### Hypothesis 1: cultural group differences

Regarding emotion regulation, as shown in Table 2, the European American group reported significantly greater trauma-specific rumination, psychological inflexibility and seeking out others for comfort and sympathy (interpersonal emotion regulation strategy of soothing) than the Asian American group. The Asian American group did not show greater self-reported emotion regulation than the European American group.

In terms of cognitive appraisals, the Asian American group reported significantly greater secondary control and cultural beliefs about adversity, and tended to report greater fatalism (pessimism) (*p* = 0.057, *d* = 0.28), than the European American group. The European American group reported greater negative cognitive appraisals about the self, negative communal self-appraisals and negative social/cultural self-appraisals than the Asian American group.

### Hypothesis 2: cultural group moderation analyses

Moderation analyses are presented in Tables 3 and 4 (correlation analyses for each cultural group are presented in Supplemental Tables 1 and 2).

**Table 3:** Summary of regression analyses for emotion regulation analyses with posttraumatic stress disorder symptoms as the outcome variable.

Predictors	B	SE	*t*	*p*	LLCI	ULCI
Difficulties in emotion regulation	0.77	0.22	3.50	< 0.001	0.34	1.21
Cultural group	− 1.67	5.89	− 0.28	0.78	− 13.28	9.93
Interaction	− 0.003	0.14	− 0.02	0.99	− 0.29	0.28
Emotional control	1.60	0.59	2.69	0.01	0.42	2.77
Cultural group	16.18	15.18	1.07	0.29	− 13.76	46.12
Interaction	− 0.56	0.40	− 1.41	0.16	− 1.35	0.23
Rumination	2.34	0.58	4.00	< 0.001	1.18	3.49
Cultural group	6.01	5.32	1.13	0.26	− 4.48	16.49
Interaction	− 0.69	0.37	− 1.89	0.06	− 1.42	0.03
Trauma− specific rumination	1.25	0.28	4.44	< 0.001	0.69	1.80
Cultural group	3.77	4.89	0.77	0.44	− 5.88	13.41
Interaction	− 0.12	0.18	− 0.67	0.50	− 0.48	0.24
Suppression	1.43	0.72	1.97	0.05	0.00	2.85
Cultural group	0.64	7.90	0.08	0.94	− 14.94	16.22
Interaction	− 0.39	0.47	− 0.83	0.41	− 1.31	0.53
Enhance positive affect	− 0.34	0.82	− 0.41	0.68	− 1.95	1.27
Cultural group	− 9.94	8.97	− 1.11	0.27	− 27.63	7.75
Interaction	0.34	0.51	0.66	0.51	− 0.66	1.34
Perspective taking	1.50	0.78	1.94	0.05	− 0.03	3.03
Cultural group	1.25	6.57	0.19	0.85	− 11.71	14.20
Interaction	− 0.44	0.48	− 0.91	0.36	− 1.39	0.51
Soothing	0.23	0.70	0.33	0.74	− 1.15	1.61
Cultural group	− 5.58	6.73	− 0.83	0.41	− 18.85	7.69
Interaction	0.19	0.44	0.43	0.67	− 0.68	1.07
Social modelling	1.84	0.79	2.33	0.02	0.28	3.41
Cultural group	8.24	7.95	1.04	0.30	− 7.42	23.91
Interaction	− 0.83	0.50	− 1.66	0.10	− 1.82	0.16

LLCI, lower level confidence interval; ULCI, upper level confidence interval.

**Table 4:** Summary of regression analyses for cognitive appraisal analyses with posttraumatic stress disorder symptoms as the outcome variable.

Predictor	B	*SE*	*t*	*p*	LLCI	ULCI
Primary control	0.76	0.29	2.59	0.01	0.18	1.34
Cultural group	3.10	6.91	0.45	0.65	− 10.52	16.72
Interaction	− 0.24	0.19	− 1.26	0.21	− 0.60	0.13
Secondary control	0.39	0.37	1.06	0.29	− 0.34	1.12
Cultural group	2.46	13.06	0.19	0.85	− 23.29	28.22
Interaction	− 0.14	0.24	− 0.59	0.56	− 0.62	0.34
Fatalism (pessimism)	3.38	1.01	3.34	0.001	1.38	5.37
Cultural group	11.51	8.86	1.30	0.20	− 5.95	28.97
Interaction	− 1.34	0.65	− 2.06	0.04	− 2.63	− 0.06
Fatalism (non− judgemental)	4.40	1.82	2.42	0.02	0.82	7.98
Cultural group	2.57	6.73	0.38	0.70	− 10.69	15.83
Interaction	− 1.20	1.17	− 1.03	0.30	− 3.50	1.10
Psychological inflexibility	1.29	0.26	5.03	< 0.001	0.78	1.79
Cultural group	4.68	4.45	1.05	0.30	− 4.10	13.45
Interaction	− 0.22	0.17	− 1.29	0.20	− 0.55	0.12
Cultural beliefs about adversity	− 0.47	0.65	− 0.72	0.47	− 1.76	0.82
Cultural group	− 4.90	14.55	− 0.34	0.74	− 33.59	23.80
Interaction	0.05	0.42	0.11	0.91	− 0.78	0.87
Negative self	0.43	0.09	4.62	< 0.001	0.24	0.61
Cultural group	2.25	4.01	0.56	0.58	− 5.66	10.15
Interaction	− 0.04	0.06	− 0.72	0.47	− 0.17	0.08
Negative world	1.07	0.28	3.74	< 0.001	0.50	1.63
Cultural group	− 1.27	5.75	− 0.22	0.83	− 12.61	10.07
Interaction	− 0.18	0.19	− 0.96	0.34	− 0.55	0.19
Self− blame	2.27	0.42	5.45	< 0.001	1.45	3.09
Cultural Group	7.83	4.26	1.84	0.07	− 0.57	16.22
Interaction	− 0.78	0.28	− 2.76	0.01	− 1.34	− 0.22
External	1.54	0.32	4.87	< 0.001	0.92	2.16
Cultural group	1.59	4.37	0.36	0.72	− 7.03	10.21
Interaction	− 0.19	0.21	− 0.91	0.36	− 0.60	0.22
Communal self	1.55	0.24	6.41	< 0.001	1.07	2.02
Cultural group	7.92	4.34	1.82	0.07	− 0.64	16.48
Interaction	− 0.31	1.6	− 1.98	0.049	− 0.61	− 0.00
Cultural/social self	1.62	0.27	5.94	< 0.001	1.08	2.15
Cultural group	5.36	4.59	1.17	0.25	− 3.70	14.42
Interaction	− 0.25	0.18	− 1.37	0.17	− 0.62	0.11

LLCI, lower level confidence interval; ULCI, upper level confidence interval.

**Figure 2:**
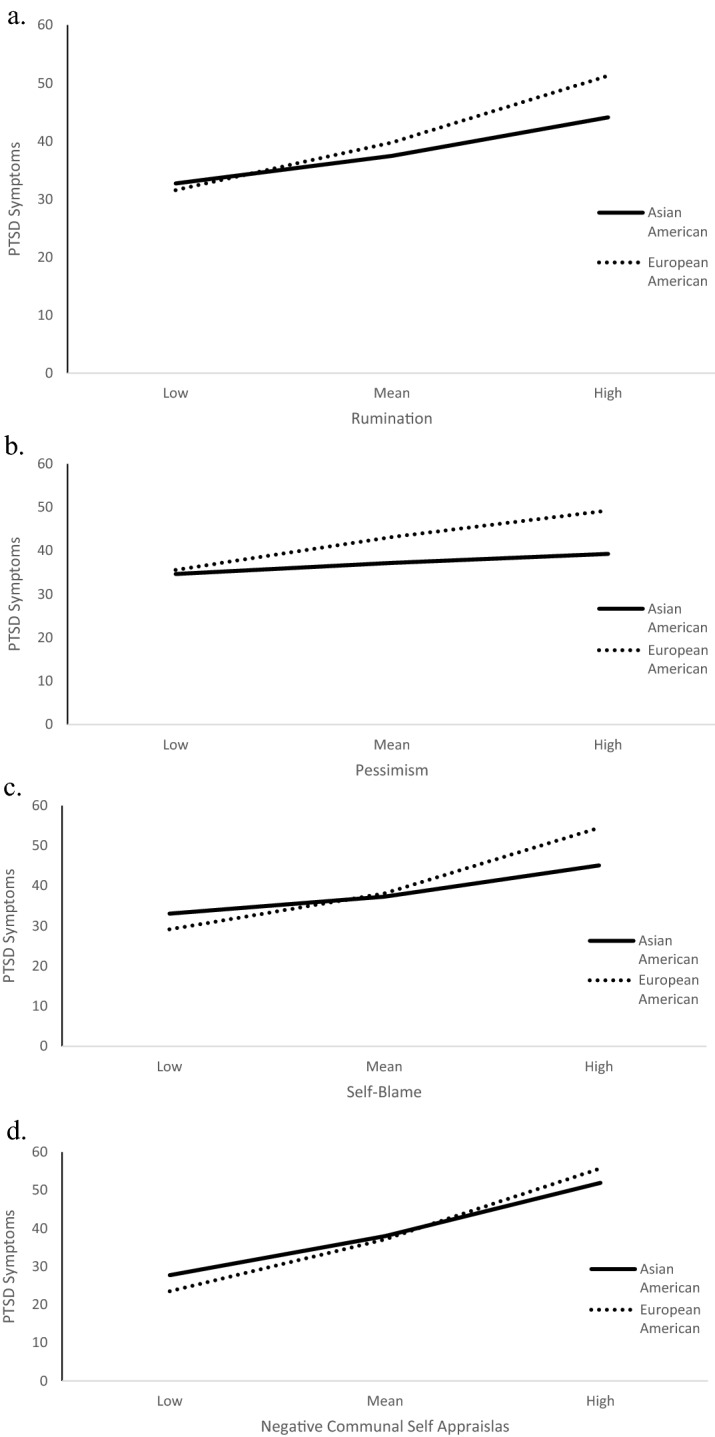
Figure depicting slimple slopes for rumination (**a**), pessimism (**b**), self-blame (**c**) and negative communal self-appraisals (**d**) moderation analyses.

Regarding emotion regulation, the moderation analysis for brooding rumination and PTSD symptoms was approaching significance, *R*^*2*^ change = 0.01, *F*(1,199) = 3.56, *p* = 0.06. Given previous cross-cultural research demonstrates cultural differences in rumination, rumination being specifically related to our hypotheses, and the effect size for the interaction term approaching moderate (*f*^*2*^ = 0.10), we conducted exploratory follow-up analyses. We found that the association between brooding rumination and PTSD symptoms was larger for the European American group, B = 1.64, *SE* = 0.27, *t* = 6.17, *p* < 0.0001, 95%CI[1.12–2.17], than the Asian American group, B = 0.95, *SE* = 0.26, *t* = 3.62, *p* < 0.001, 95%CI[0.43–1.47]. Additionally, while the two cultural groups had similar levels of PTSD symptomatology at low levels of rumination, at high levels of rumination the European American group had higher levels of PTSD symptomatology than the Asian American group (Fig. 2a). There was no evidence that cultural group moderated the associations between the other emotion regulation approaches and PTSD symptoms (see Table 3 and Supplementary Material).

Regarding cognitive appraisals, cultural group moderated the associations between fatalism (pessimism) and PTSD symptoms, *R*^*2*^ change = 0.02, *F*(1,199) = 4.24, *p* = 0.04 (Fig. 2b), self-blame and PTSD symptoms, *R*^*2*^ change = 0.02, *F*(1,199) = 7.62, *p* < 0.01 (Fig. 2c), and negative communal self-appraisals and PTSD symptoms, *R*^*2*^ change = 0.01, *F*(1,199) = 3.92, *p* = 0.049 (Fig. 2d). The association between fatalism (pessimism) and PTSD symptoms was significant for the European American group, B = 2.03, *SE* = 0.45, *t* = 4.52, *p* < 0.001, 95%CI[1.15–2.92], but not for the Asian American group, B = 0.69, *SE* = 0.48, *t* = 1.42, *p* = 0.16, 95%CI[− 0.27 to 1.64]. The associations between both self-blame and negative communal self-appraisals and PTSD symptoms were large for the European American group (self-blame B = 1.49, *SE* = 0.18, *t* = 48.34, *p* < 0.001, 95%CI[1.14–1.84]; negative communal self-appraisals, B = 1.24, *SE* = 0.11, *t* = 11.53, *p* < 0.001, 95%CI[1.03–1.45]), but weaker for the Asian American group (self-blame, B = 0.71, *SE* = 0.23, *t* = 3.14, *p* = 0.002, 95%CI[0.26–1.15]; negative communal self-appraisals, B = 0.93, *SE* = 0.11, *t* = 8.10, *p* < 0.001, 95%CI[0.70–1.16]). There was no evidence that cultural group moderated the associations between the other cognitive appraisal types and PTSD symptoms (see Table 4 and Supplementary Material).

### Hypothesis 3: mediation analyses

We found that there was a significant indirect pathway between independent self-construal and PTSD symptoms through emotional control, *B* = 0.07, *SE* = 0.03, 95%CI[0.03–0.13], psychological inflexibility, *B* = − 0.11, *SE* = 0.05, 95%CI[− .21 to − .01], brooding rumination, *B* = − 0.07, *SE* = 0.04, 95%CI[− .15 to − .001], interpersonal emotion regulation, *B* = 0.05, *SE* = 0.02, 95%CI[0.01–0.10], difficulties in emotion regulation, *B* = − 0.13, *SE* = 0.05, 95%CI[− .23 to − .03], primary control appraisals, *B* = 0.08, *SE* = 0.03, 95%CI[0.03–0.14], negative self-appraisals, *B* = − 0.16, *SE* = 0.05, 95%CI[− .27 to − .06], and cultural beliefs about adversity, *B* = − 0.06, *SE* = 0.03, 95%CI[− .12 to − .003].

There was a significant indirect pathway between interdependent self-construal and PTSD symptoms through emotional control, *B* = 0.05, *SE* = 0.03, 95%CI[0.003 to 0.12], interpersonal emotion regulation, *B* = 0.05, *SE* = 0.03, 95%CI[0.003 to 0.12], primary control appraisals, *B* = 0.09, *SE* = 0.03, 95%CI[0.04 to 0.15], fatalism appraisals, *B* = 0.08, *SE* = 0.03, 95%CI[0.03 to 0.15], cultural beliefs about adversity, *B* = − 0.04, *SE* = 0.02, 95%CI[− .09 to − .005], and cognitive appraisals about communal and social aspects of self, *B* = 0.21, *SE* = 0.05, 95%CI[0.11 to 0.31].

### Exploratory analyses: moderated mediation analyses

There was evidence that cultural group moderated the indirect effects of independent self-construal on PTSD symptoms through psychological inflexibility, index = 5.38, *SE* = 2.03, 95%CI[1.20–9.20], brooding rumination, index = 4.67, *SE* = 1.44, 95%CI[1.72–7.48], and public and communal self-appraisals, index = 5.00, *SE* = 2.41, 95%CI[0.22–9.74]. In each instance, the indirect effect was significant for the European American group (psychological inflexibility, B = − 5.57, *SE* = 1.38, 95%CI[− 8.10 to − 2.64], brooding rumination B = − 4.05, *SE* = 1.05, 95%CI[− 6.22 to −2.10], public and communal self-appraisals, B = − 3.86, *SE* = 1.80, 95%CI[− 7.22 to − 0.17]), but not the Asian American group (psychological flexibility B = − 0.20, *SE* = 1.54, 95%CI[− 3.17 to 2.82], brooding rumination, B = 0.62, *SE* = 1.09, 95%CI[− 1.70 to 2.58], public and communal self-appraisals, B = 1.14, *SE* = 1.65, 95%CI[- 2.05 to 4.45]). Cultural group also moderated the indirect effect of interdependent self-construal on PTSD symptoms through fatalism appraisals, index = − 2.02, *SE* = 1.08, 95%CI[− 4.33 to − 0.02], with the indirect effect being significant for the European American group, B = 2.82, *SE* = 0.89, 95%CI[1.32 to 4.80], but not the Asian American group, B = 0.80, *SE* = 0.82, 95%CI[− 0.60 to 2.62].

## Discussion

This study aimed to investigate whether the associations between both emotion regulation and cognitive appraisals and PTSD symptoms differed between Asian American and European American trauma survivors. Regarding Hypothesis 1, the European American group reported greater trauma-specific rumination, psychological inflexibility, seeking out others for comfort and negative self-appraisals than the Asian American group. The Asian American group reported greater secondary control appraisals, cultural beliefs about adversity and fatalism (pessimism) than the European American group. In support of Hypothesis 2, the associations between rumination and PTSD symptoms and between self-blame and PTSD symptoms were large for the European American group, but weaker for the Asian American group. In contrast to our hypothesis, the associations between fatalism (pessimism) and negative communal self-appraisals and PTSD symptoms were also larger for the European American group than the Asian American group. Finally, there was mixed support for Hypothesis 3. We found that, as predicted, there was a significant indirect pathway between independent self-construal and PTSD symptoms through psychological inflexibility (European American group only), brooding rumination (European American group only), primary control appraisals and negative self-appraisals. There was also a significant indirect pathway between interdependent self-construal and PTSD symptoms through emotional control, interpersonal emotion regulation, fatalism appraisals (European American group only), cultural beliefs about adversity, and cognitive appraisals about communal and social aspects of self. However, contrary to that predicted, we found a significant indirect pathway between independent self-construal and PTSD symptoms through emotional control, cultural beliefs about adversity and interpersonal emotion regulation, which were proposed to be associated with interdependent self-construal. We also found a significant indirect pathway between interdependent self-construal and PTSD symptoms through primary control appraisals, which we predicted would be more associated with independent self-construal.

Our cultural group difference findings support notions that those from Asian cultural backgrounds place greater value on cognitive appraisals of secondary control, fatalism, and specific cultural beliefs about adversity^7,32,33,38^ and those from European cultures emphasize their self-aspects^29,42^. As the foundations of psychological flexibility are associated with Eastern ideologies^23^, it is not surprising that the European American group reported less psychological flexibility. Additionally, cross-cultural research indicates that European Americans are more likely to seek out and value explicit support than Asian Americans^43^. Thus, in the context of PTSD, Asian Americans may report being less likely to seek out others for comfort. Finally, while our findings contradict previous research showing that rumination is greater among Asian Americans than European Americans^21^, previous research has focused on college students. Comparatively, as our participants were trauma survivors with PTSD symptomology, our finding may reflect that rumination is more pertinent for psychological adjustment among European Americans in a more general population^20,21^.

Our brooding rumination moderation findings, whilst somewhat exploratory as findings were only approaching significance, support the notion that European Americans tend to suffer worse outcomes of brooding rumination when compared with Asian Americans^20,21^. It has been proposed that those from Asian cultures are less likely to get “stuck” in negative emotional content as they view negative emotions as less problematic and more transient, and are more likely to self-distance from their emotional experiences^20,44^. Our self-blame moderation findings replicated Bernardi and Jobson^36^, who similarly found that the association between self-blame and PTSD symptoms was significantly weaker for Asian Australians than European Australians. Bernardi and Jobson claimed that self-blame may be associated with greater PTSD symptomatology among Western trauma survivors as these cultures value mastery and responsibility and hence attributing more individual responsibility for the trauma is associated with greater PTSD symptomatology. We predicted that the associations between fatalism and negative communal self-appraisals would be more pertinent to the posttrauma recovery of Asian Americans. However, we found that these associations were stronger for the European American group. We are not certain as to why this was the case. It is possible that for European Americans fatalism and negative communal self-appraisals are less common in this cultural context, and as such less protective in relation to PTSD symptoms. If this is the case, there are potentially multiple pathways and mechanisms by which culture may exert an influence on PTSD symptoms. As these are all relatively new factors being studied in the area of culture and PTSD, further research is needed.

There was some evidence to support the notion that cultural values (i.e., independent and interdependent self-construal) are associated with cognitive appraisal and emotion regulation processes that in turn are associated with PTSD symptoms. We found that there was a significant indirect pathway between independent self-construal and PTSD symptoms through cognitive appraisal and emotion regulation processes proposed to be associated with independence; psychological inflexibility, brooding rumination, primary control appraisals, and negative self-appraisals. We also found that that there was a significant indirect pathway between interdependent self-construal and PTSD symptoms through cognitive and emotion regulation processes proposed to be associated with interdependence; emotional control, interpersonal emotion regulation, fatalism appraisals, cultural beliefs about adversity, and cognitive appraisals about communal and social aspects of self. However, our findings indicated that some cognitive appraisals (e.g., primary control) and emotion regulation approaches (e.g., emotional control) were associated with both aspects of self-construal and some cognitive appraisals and emotion regulation approaches did not align with that predicted. Moreover, cultural group moderated some of the indirect effects, whereby the indirect effects were significant for the European American group but not the Asian American group. These findings highlight that including self-construal provides a more complex story and supports emerging research indicating the importance of considering both cultural group and cultural values when furthering cross-cultural PTSD research^36,45^.

PTSD theoretical models and treatments emphasize the role of cognitive appraisals and emotion regulation. This study highlights that greater understanding is needed regarding cultural influences on the processes known to underpin posttrauma recovery. There also needs to be greater examination of the role of individual cultural values in PTSD to further guide how psychological interventions for PTSD can be culturally tailored. Huey et al.^6^ found that culturally tailoring psychological interventions for Asian Americans can enhance treatment outcomes. For over a decade now, researchers have identified the urgency of being able to culturally-adapt psychological interventions to better meet the needs of Asian Americans^45^. This study contributes to the needed evidence to guide the cultural tailoring of PTSD interventions for Asian Americans. This is particularly pertinent for emotion regulation and trauma-related cognitive appraisals, which are often key clinical targets in first-line PTSD psychological interventions.

Some preliminary implications of the findings include that the static categorization of emotion regulation strategies as either putatively adaptive or maladaptive may not generalize to Asian American trauma survivors. Additionally, considerations of both a client's cultural background (heritage) and idiosyncratic self-construal may be important factors in guiding treatment selection and implementation. Currently, there is little understanding as to how culture influences mental health. Culture may affect the types of strategies that are useful in reducing or perpetuating distress. For instance, if people hold emotion regulation and cognitive appraisal tendencies that do not accord with their surrounding sociocultural environment, does this serve to protect or diminish mental health^46^? Thus, the wider sociocultural context needs to be considered.

There are some limitations. First, the study was cross-sectional and thus, causality cannot be inferred. Second, the study included a mTurk sample. Thus, the generalizability of our findings needs to be considered. We included several quality assurance checks in our survey (response validity indicators^47,48^, Phase 1 screened for trauma and cultural background, our cultural inclusion criteria were strictly applied) to ensure the validity of our findings. Additionally, previous research shows that mTurk samples provide high quality data^49,50^ and are more representative than student/university samples or alternate internet-based samples^51^. Thus, we have confidence in our results, but further replication studies are needed. Third, the study was not a clinical sample. Nevertheless, over 50% of each cultural group met provisional diagnosis for PTSD. Fourth, while the study was adequately powered for the moderation analyses, a larger sample would benefit the moderated mediation analyses. Fifth, as noted in the Introduction, the literature highlights the bi-directional relationships between cognitive appraisals and emotion regulation in the context of PTSD^16,17^. It was beyond the scope of this study to examine these pathways, particularly given the sample size. However, future research is needed to examine the influences of culture on these relationships in PTSD and to consider modelling studies to examine the relationship between different emotion regulation approaches and cognitive appraisals. Additionally, emotion regulation and cognitive appraisals are broad, complex and non-static processes. While we examined emotion regulation and cognitive appraisals frequently focused on in the PTSD literature, moving forward future research needs to unpack the complexities of these constructs. Sixth, while the expected cultural differences were observed in independent self-construal, there were no cultural group differences in interdependent self-construal. This further highlights the need to also examine cultural values at the individual level. Seventh, to better address health disparities there is a need to focus on smaller-sized subgroup populations within Asian Americans^54^. As this study was one of the first studies to investigate these variables in the context of PTSD among Asian Americans, we adopted the approach of past researchers and focused on Asian Americans as a group^21,43^. However, while all participants identified as Asian American and reported both parents and all four grandparents being of Asian heritage, there was heterogeneity in terms of country of birth, migration status (first- or second-generation migrant) and time living in the US. Thus, as this research area moves forward there is a need to explore these concepts among subgroup populations and greater exploration of cultural values (e.g., acculturation)^54^. Finally, while the groups did not differ in identified index traumas and we included time since trauma and trauma type as covariates in our analyses, trauma type (e.g., interpersonal, childhood) may have influenced findings.

In conclusion, the European American group reported significantly greater trauma-specific rumination, psychological inflexibility, seeking out others for comfort and sympathy and negative self-cognitive appraisals than the Asian American group. The Asian American group reported significantly greater cognitive appraisals of secondary control, cultural beliefs about adversity and fatalism than the European American group. Second, the associations between rumination, self-blame, pessimism and negative communal self- appraisals and PTSD symptoms were larger for the European American group than the Asian American group. Third, there was evidence for indirect pathways between self-construal and PTSD symptoms through certain cognitive appraisals and emotion regulation, and cultural group moderated several of these indirect effects. Taken together, our findings provide promising initial indications of the influence of culture on key psychological mechanisms underpinning PTSD symptomology, and highlight fruitful avenues for further research, including with clinical populations.

## Methods

### Design

The study obtained ethical approval from the Monash Human Research Ethics Committee (29651). Research was performed in accordance with the Declaration of Helsinki. The study employed a cross-sectional design.

### Participants

To determine our target sample size, we used G*Power 3.1. Our estimates were based on the moderation analyses. We used small to moderate effect sizes^19,36^, an alpha of 0.05, and 80% power. It was estimated that the study required 101 participants per cultural group.

We used TurkPrime^52^ to recruit adults from the United States on Amazon’s Mechanical Turk platform. Participants received US$6 for completing the study. Inclusion criteria were: (a) having experienced a criterion A trauma experience (as indexed by the Life Events Checklist), (b) residing in the US and identifying as having European heritage (i.e., participant, both parents and all four grandparents had to be of European heritage) or Asian heritage (i.e., participant, both parents and all four grandparents had to be of Asian heritage), (c) being over 18 years of age, and (d) able to complete the online survey in English. Exclusion criteria included rapid responders (i.e., those who completed the survey in < 15 min), and scoring below the conscientious response cut-off (those who did not score a minimum of three correct responses on the Conscientious Responder Scale^53^). To ensure quality control, additional mTurk inclusion criteria were: participants had to have completed at least 100 Human Intelligence Tasks (HITs) and had to have a HIT approval ratio (HAR) of at least 95%.

Participants were invited to complete a Phase 1 screener, which screened for trauma exposure and cultural background. Around 600 participants were screened and those who met our inclusion criteria for trauma exposure and cultural background (*n* = 344) were invited to complete Phase 2; the empirical study. In total, 228 people completed the study, of which, we excluded 21 participants from the analyses according to the following exclusion criteria: no trauma exposure (*n* = 8), rapid responder (*n* = 2), and cultural background did not meet inclusion criteria (*n* = 11). The final sample consisted of 207 trauma survivors (European American *n* = 104; Asian American *n* = 103).

### Measures

#### Trauma exposure and symptomatology

##### PTSD checklist for the DSM-5 with life events checklist (PCL-5)^55^

The LEC screens for life-time exposure to potentially traumatic events. In Phase 1, to reduce demand characteristics, items from the LEC were included within a list of other lifetime events (positive, negative, neutral) and participants were requested to select events that they had experienced. In Phase 2, the LEC was included to assess details about participant’s index trauma (trauma type, time since trauma).

The PCL-5 is a 20-item self-report measure of PTSD symptoms, with items scored on 5-point Likert-type scales. Participants completed the PCL-5 in response to the index trauma reported on the LEC. A total PTSD severity score is calculated by summing the responses and can range from 0 to 80, with higher scores indicating greater PTSD symptom severity^55^. A PCL-5 cut-point score of 33 has been suggested as indicating a provisional PTSD diagnosis^55^. The PCL-5 has good psychometric properties and is used in cross-cultural PTSD research^55^. In this study, the PCL-5 had excellent internal consistency; European American group (McDonald’s Omega = 0.97) and Asian American group (McDonald’s Omega = 0.96).

##### Hospital Anxiety and Depression Scale (HADS)

The HADS^56^ was used to assess depression (7 items) and anxiety (7 items) symptoms. We assessed symptoms of depression and anxiety in order to provide greater details regarding symptomatology. Items were scored on 4-point Likert-type scales and item scores were summed to provide a total depression and anxiety score, with higher scores indicating greater symptom severity. The HADS has good validity and reliability, including in cross-cultural research^57^. In this study internal consistency was good (European American McDonald’s Omega = 0.88, 0.83; Asian American McDonald’s Omega = 0.86, 0.84, for anxiety and depression, respectively).

#### Emotion regulation measures

##### Difficulties in emotion regulation (DERS)^10^

The DERS is a 36-item self-report measure of emotion regulation problems. Responses are provided on 5-point Likert-type scales and scores are summed to present a total score of emotion regulation problems, with higher scores indicating greater difficulties. Validity and reliability of the DERS are good^10^. In the current study, internal consistency was excellent; European American group (McDonald’s Omega = 0.94) and Asian American group (McDonald’s Omega = 0.93).

##### Emotion control values (ECV)^58^

The ECV is a 6-item self-report measure that assesses general beliefs and values about controlling one’s emotions. Participants rated their agreement with the item statements on 11-point Likert-type scales, with three items being reverse scored. Higher scores indicated greater value placed in controlling one’s emotions. The ECV was developed for use in cross-cultural research with European and Asian populations, with the original studies demonstrating good reliability^58^. In the current study, internal consistency was good; European American group (McDonald’s Omega = 0.75) and Asian American group (McDonald’s Omega = 0.75).

##### Repetitive thinking questionnaire—10 (RTQ-10)^59^

The RTQ-10 is a 10 item self-report questionnaire measuring repetitive negative thinking. While the original RTQ-10 asks participants to rate how true the given statements are when they feel distressed or upset, for the current study, items were asked with respect to the participant’s index trauma. Participants rated how true each statement was on 5-point Likert-type scales with higher scores reflecting greater trauma-specific rumination. In the current study, internal consistency was excellent; European American group (McDonald’s Omega = 0.94) and Asian American group (McDonald’s Omega = 0.94).

##### Ruminative response scale-short form (RRS-SF)^60^

Brooding rumination was measured using the brooding subscale of the RRS-SF (RRS-B ^60^). The five items were scored on 4-point Likert-type scales. Item scores were summed, with higher scores indicating greater levels of brooding rumination. The RRS-B has been shown to have good psychometric properties, including in cross-cultural research^61^. Here the RRS-B demonstrated good internal consistency (European American group McDonald’s Omega = 0.86; Asian American group McDonald’s Omega = 0.89).

##### Emotion regulation questionnaire (ERQ)^22^

We assessed expressive emotional suppression using the ERQ. The ERQ includes 4 items that assess expressive suppression and items are responded to on 7-point Likert-type scales. The ERQ is a routinely used measure of emotion suppression, including in cross-cultural research^19^, and has good psychometric properties^22^. In the current study, internal consistency was good (European American McDonald’s Omega = 0.70; Asian American McDonald’s Omega = 0.73).

##### Interpersonal emotion regulation questionnaire (IERQ)^62^

The IERQ contains four subscales (5 items each which are responded to on 5-point Likert-type scales); enhancing positive affect (i.e., tendency to seek out others to increase feelings of happiness and joy), perspective taking (i.e., involves the use of others to be reminded not to worry and that others have it worse), soothing (i.e., consists of seeking out others for comfort and sympathy) and social modelling (i.e., involves looking to others to see how they might cope with a given situation). The questionnaire has good psychometric properties^20^. In the current study, internal consistency was excellent (European American McDonald’s Omega = 0.95, Asian American McDonald’s Omega = 0.95).

##### Acceptance and action questionnaire—second version (AAQ-II)^63^

The AAQ-II is a 7-item questionnaire frequently used to assess psychological flexibility/inflexibility. It measures altering unwanted thoughts/feelings and the inability to persist through present thoughts/feelings without needless defence. Participants rated how true each statement was on 7-point Likert scales, with higher scores indicating greater inflexibility. In the current study, internal consistency was excellent (European American McDonald’s Omega = 0.96, Asian American McDonald’s Omega = 0.95).

#### Appraisal measures

##### Primary-Secondary Control Scale (PSCS)^64^

The PSCS is a 37-item self-report questionnaire that assesses cognitive appraisals of primary (17 items) and secondary (20 items) control in relation to an adverse life event; the index trauma. Responses were made on 4-point Likert-type scales and are summed for each subscale, with higher scores indicating greater degree of control beliefs. The PSCS has good psychometric properties, including in cross-cultural samples^64^. In the current study, internal consistency was excellent (European American McDonald’s Omega = 0.95, Asian American McDonald’s Omega = 0.95).

##### Posttraumatic Cognitions Inventory (PTCI)^65^

The PTCI is a 33-item measure that assesses trauma-related cognitive appraisals. It consists of three subscales; negative self, negative world and perceived self-blame regarding the trauma. The PTCI has good psychometric properties^65^ and has been used in cross-cultural research^36^. In the current study the PTCI demonstrated excellent internal consistency (European American McDonald’s Omega = 0.98; Asian American McDonald’s Omega = 0.96).

##### Fatalism questionnaire^66,67^

The Fatalism Questionnaire includes six items that assess an individual’s propensity to believe that one’s destiny is externally determined. It has good psychometric properties^66,67^ and has been used cross-culturally ^66,67^. In the current study, internal consistency was good (European American McDonald’s Omega = 0.86; Asian American McDonald’s Omega = 0.86).

##### Chinese Cultural Beliefs about Adversity Scale (CBA)^33^

The CBA assesses specific cultural beliefs about adversity. It contains nine items; seven items focus on positive cultural beliefs about adversity and two items focus on negative cultural beliefs about adversity (reversed scored). Respondents indicate the degree to which they agree with each item on 6-point Likert-type scales. Higher scores indicate a higher degree of agreement with positive cultural beliefs about adversity. Psychometric properties are good^24^. In the current study, internal consistency was good (European American McDonald’s Omega = 0.77; Asian American McDonald’s Omega = 0.82).

##### Public and Communal Self-Appraisals Measure (PCSAM)^35^

The PCSAM is a 21-item self-report measure that assesses cognitive appraisals of the impact of trauma on public and communal aspects of self. It is comprised of three subscales: external appraisals (challenges to beliefs and belonging) (7 items), communal aspects of self (7 items), and cultural/social roles and identity (7 items). Participants rated their agreement with each statement on a 7-point Likert scale. The PCSAM has been used previously in cross-cultural research and has demonstrated good reliability^35^. In the current study, internal consistency was good (European American McDonald’s Omega = 0.96, Asian American McDonald’s Omega = 0.94).

#### Self-construal measure

##### Self-construal scale (SCS)^34^

The SCS is a 30-item scale that assesses how people view themselves in relation to others. It is comprised of two subscales; independent self-construal (15 items) and interdependent self-construal (15 items). Participants respond to 30 self-statements on 7-point scales and scores are totaled providing an independent and interdependent score. This scale is widely used in cross-cultural research^34^. In the current study, internal consistency was good (independent self-construal European American McDonald’s Omega = 0.78, Asian American McDonald’s Omega = 0.82; interdependent self-construal European American McDonald’s Omega = 0.83, Asian American McDonald’s Omega = 0.88).

### Procedure

Participants who met eligibility criteria in Phase 1 were invited to complete Phase 2; an online survey hosted on Qualtrics. At the commencement of the survey participants were provided with an explanatory statement and participants provided informed consent by commencing the survey. We also inserted items from the Conscientious Responder Scale^53^; a scale developed to differentiate between conscientious and indiscriminate responses on a survey.

### Data analysis plan

Prior to hypothesis-testing, data cleaning was conducted using Microsoft Excel. All subsequent analyses were conducted using IBM SPSS Statistics 27. As several variables were not normally distributed and transformations did not improve normality, bootstrapping (5000 bootstrapped samples) were used for all analyses. Due to group differences in age and education and potential influences of trauma type and time since trauma on findings[e.g., ^68,69^, these variables were included as covariates in all analyses. Following current recommendations^70^, anxiety and depression were not included as covariates but were rather used to provide further details about the sample.

To assess Hypothesis 1, group differences were explored using a series of one-way (Asian American vs. European American) Analysis of Covariances, with cognitive appraisals and emotion regulation the dependent variables. For the variables, interpersonal emotion regulation, trauma-related cognitions and public and communal self-cognitive appraisals Multivariate Analysis of Covariances were used with the sub-scales as dependent variables. To examine Hypothesis 2, we conducted a series of moderation analyses for each appraisal and emotion regulation type using PROCESS (model 1)^71^. We also conducted exploratory analyses using regression analyses to examine the associations between the (a) emotion regulation variables and PTSD symptoms, and (b) cognitive appraisal variables and PTSD symptoms for each cultural group. Given our sample size, these analyses were exploratory and are reported in Supplementary Material. To test Hypothesis 3, we conducted a series of mediation analyses using PROCESS (model 4)^71^ with self-construal as the predictor, PTSD symptoms as the dependent variable, and the mediators were cognitive appraisals and emotion regulation. For our exploratory analyses, a series of moderated mediation models were tested using bootstrapping (“PROCESS” macro, model 7)^71^ with bias-corrected 95% confidence intervals to assess the significance of the indirect effects at differing levels of the moderator (cultural group). Confidence intervals were used to determine significance of results, with confidence intervals not including 0 being considered significant.

## Supplementary Information

Supplementary Information.

## Data Availability

The datasets generated and analysed during the current study are available in the OSF repository, https://osf.io/zkwna/.
