# Supplementary material for: Investigating the associations between cognitive appraisals, emotion regulation and symptoms of posttraumatic stress disorder among Asian American and European American trauma survivors
Source: Sci Rep. 2022 Oct 28;12:18127. doi: 10.1038/s41598-022-22995-3 (PMC9616820; doi:10.1038/s41598-022-22995-3)
Supplement: Supplementary file 1 — Supplementary Information. [file 41598_2022_22995_MOESM1_ESM.docx]

**Supplementary Material**

**Supplemental Table 1**

Partial Correlation Analyses [with 95% Confidence Intervals] for Emotion Regulation for the Asian American Group (top half of table) and for the European American Group (lower half of table)

|  | 1 | 2 | 3 | 4 | 5 | 6 | 7 | 8 | 9 | 10 | 11 |
| --- | --- | --- | --- | --- | --- | --- | --- | --- | --- | --- | --- |
| 1. PTSD Symptoms | 1.00 | .61***  [.43-.74] | .12  [-.07-.29] | .22*  [.02-.41] | .24*  [.05-.43] | .08  [-.11-.28] | .15  [-.06-.32] | .64***  [.46-.77] | .34***  [.19-.51] | .19*  [.02-.36] | .56***  [.41,.69] |
| 2. Difficulties in Emotion Regulation | .57***  [.40,.72] | 1.00 | .07  [-.12, .24] | .20*  [-.03, .42] | .25*  [.04,.44] | .11  [-.11,.32] | .09  [-.14,.29] | .58***  [.41,.71] | .52***  [.36,.65] | .36***  [.17,.53] | .78***  [.68,.86] |
| 3. Positive Affect | .01  [-.21,.20] | -.05  [-.25,.15] | 1.00 | .51***  [.34,.65] | .59***  [.43,.72] | .64***  [.50,.77] | .10  [-.14,.32] | .001  [-.17,.20] | .08  [-.10,.27] | -.36***  [-.53,-.17] | .01  [-.19,.20] |
| 4. Perspective Taking | .27**  [.08,.46] | .12  [-.07,.34] | .52***  [.36,.66] | 1.00 | .67***  [.51,.79] | .67*** [.53,.78] | .17  [-.08,.38] | .17  [-.03,.37] | .08  [-.11,.29] | -.10  [-.34,.12] | .17  [-.03,.38] |
| 5. Soothing | .12  [-.09,.32] | .16  [-.01,.33] | .01***  [.48,.72] | .53***  [.35,.68] | 1.00 | .66***  [.53,.77] | .02  [-.20,.23] | .17  [-.03,.37] | .17  [-.03,.35] | -.27*  [-.48,-.05] | .22*  [.02,.41] |
| 6. Social Modelling | .27**  [.03,.47] | .16  [-.02,.34] | .65***  [.51,.76] | .75***  [.66,.83] | .64***  [.48,.79] | 1.00 | .09  [-.14,.31] | .04  [-.16,.26] | .18  [-.01,.38] | -.13  [-.36,.12] | .08  [-.14,.28] |
| 7. Emotional Control | .35***  [.14,.55] | .32**  [.11,.50] | .14  [-.02,.29] | .32**  [.12,.50] | .18  [.00,.35] | .16  [-.02,.33] | 1.00 | .08  [-.12,.26] | .07  [-.17,.29] | .11  [-.15,.33] | .05  [-.16,.26] |
| 8. Trauma-Specific Rumination | .64***  [.47,.76] | .53***  [.37,.66] | -.01  [-.24,.22] | .13  [-.06,.34] | .22*  [.00,.40] | .12  [-.10,.34] | .29**  [.10,.47] | 1.00 | .51***  [.36,.64] | .31**  [.23,.47] | .70***  [.59,.79] |
| 9. Rumination | .51***  [.32,.68] | .62***  [.47,.74] | -.12  [-.34,.11] | -.09  [-.30,.15] | .15  [-.07,.37] | -.01  [-.24,.24] | .20*  [.01,.39] | .67***  [.54,.77] | 1.00 | -.003  [-.23,.25] | .26**  [.09,.44] |
| 10. Expressive Suppression | .28**  [.09,.48] | .27*  [.06,.45] | -.34***  [-.53,-.13] | -.08  [-.32,.16] | -.36***  [-.52,-.16] | -.28**  [-.47,-.05] | .16  [-.05,.36] | .31**  [.14,.50] | .22*  [-.01,.43] | 1.00 | .40***  [.24,.55] |
| 11. Psychological Inflexibility | .68***  [.53,.81] | .77***  [.68,.85] | -.08  [-.31,.15] | .05  [-.17,.26] | .19  [-.02,.38] | .13  [-.14,.34] | .25*  [.06,.41] | .77***  [.67,.83] | .69***  [.55,.81] | .24*  [.04,.44] | 1.00 |

*Note: ***p* < .001, ** *p* < .01, * *p* < .05

**Supplemental Table 2**

Partial Correlation Analyses for Appraisals for the Asian American Group (top half of table) and for the European American Group (lower half of table)

|  | 1 | 2 | 3 | 4 | 5 | 6 | 7 | 8 | 9 | 10 | 11 | 12 |
| --- | --- | --- | --- | --- | --- | --- | --- | --- | --- | --- | --- | --- |
| 1. PTSD Symptoms | 1.00 | .24*  [.03,.41] | .07  [-.16,.26] | .14  [-.05,.32] | .24*  [.02,.45] | -.12  [-.31,.05] | .57***  [.38,.72] | .46***  [.31,.61] | .28*  [.09,.47] | .62***  [.46,.76] | .63***  [.48,.76] | .59***  [.41,.72] |
| 2. Primary Control | .34**  [.16,.53] | 1.00 | .69***  [.58,.79] | .08  [-.15,.28] | -.01[-.21,.21] | .17  [-.05,.40] | .05  [-.14,.24] | .14  [-.07,.31] | .25*  [.05,.43] | .28*  [.09,.46] | .26*  [.06,.45] | .30**  [.08,.49] |
| 3.Secondary Control | .13  [-.06,.34] | .68***  [.57,.78] | 1.00 | .18  [-.05,.39] | -.06[-.26,.16] | .33**  [.13,.53] | -.21*  [-.39,-.01] | .03  [-.18,.23] | .02  [-.18,.20] | .01  [-.18,.23] | .02  [-.17,.21] | .05  [-.15,.23] |
| 4. Pessimism | .39***  [.21,.55] | .32**  [.15,.49] | .27*  [.12,.43] | 1.00 | .61***[.47,.73] | .10  [-.12,.30] | .19*  [.01,.36] | .17  [-.05,.40] | -.03  [-.24,.18] | .23*  [.02,.43] | .15  [-.08,.38] | .13  [-.08,.33] |
| 5. Non-judgement | .34***  [.18,.49] | .28*  [.11,.48] | .12  [-.07,.31] | .70***  [.58,.78] | 1.00 | -.16  [-.35,.07] | .41***  [.23,.58] | .13  [-.08,.35] | .16  [-.06,.36] | .30**  [.09,.50] | .22*  [.01,.42 | .26*  [.03.46] |
| 6. Cultural Beliefs about Adversity | -.15  [-.38,.08] | .28*  [.08,.44] | .39***  [.18,.55] | -.01*  [-.19,.18] | -.12[-.26,.06] | 1.00 | -.21*  [-.40,-.01] | .06  [-.16,.25] | -.01  [-.21,.18] | -.11  [-.29,.09] | -.06  [-.26,.12] | -.05  [-.25,.15] |
| 7. Negative Self | .68***  [.53,.81] | .17  [-.02,.39] | .001  [-.20,.23] | .30**  [.09,.47] | .24*[.07,.40] | -.29**  [-.51,-.05] | 1.00 | .55***  [.42,.67] | .43***  [.26,.60] | .60***  [.43,.73] | .67***  [.53,.80] | .65***  [.48,.77] |
| 8. Negative World | .56***  [.40,.69] | .08  [-.12,.29] | -.001  [-.21,.22] | .31**  [.11,.47] | .24*[.06,.41] | -.18  [-.38,.04] | .78***  [.71,.85] | 1.00 | .29**  [.08,.48] | .43***  [.27,.59] | .57***  [.44,.68] | .50***  [.36,.64] |
| 9. Self-Blame | .65***  [.53,.77] | .37***  [.19,.51] | .14  [-.08,.33] | .31**  [.12,.49] | .27*[.08,.47] | .02  [-.17,.18] | .68***  [.55,.79] | .55***  [.39,.68] | 1.00 | .32**  [.09,.51] | .41***  [.21,.57] | .36***  [.13,.55] |
| 10. External | .66***  [.54,.77] | .28**  [.09,.49] | .10  [-.09,.31] | .31**  [.11,.50] | .30**[.13,.47] | -.22*  [-.45,.02] | .74***  [.63,.84] | .60***  [.45,.73] | .52***  [.35,.66] | 1.00 | .79***  [.70,.87] | .65***  [.52,.76] |
| 11. Communal | .75***  [.66,.83] | .27*  [.07,.48] | .08  [-.12,.29] | .33**  [.13,.50] | .31**[.13,.46] | -.19  [-.43,.05] | .82***  [.74,.89] | .70***  [.57,.80] | .65***  [.52,.77] | .82***  [.72,.88] | 1.00 | .80***  [.70,.87] |
| 12. Cultural/Social | .77***  [.67,.85] | .29**  [.09,.48] | .15  [-.04,.35] | .24*  [.04,.44] | .15[-.03,.35] | -.15***  [-.37,.09] | .81***  [.72,.89] | .66***  [.54,.77] | .68***  [.58,.78] | .74***  [.60,.85] | .87***  [.83,.91] | 1.00 |

*Note: ***p* < .001, ** *p* < .01, * *p* < .05

**Moderation Analyses**

**Emotion regulation.** There was no evidence that cultural group moderated the associations between any the following emotion regulation strategies and PTSD symptoms; difficulties in emotion regulation, *R^2^* change <.001, *F*(1,199) <.001, *p* = .99, emotional control, *R^2^* change =.01, *F*(1,199) =1.98, *p* = .16, trauma-specific rumination, *R^2^* change =.001, *F*(1,199) =.45, *p* = .50, suppression, *R^2^* change =.003, *F*(1,199) =.69, *p* = .41, positive affect, *R^2^* change =.002, *F*(1,199) =.45, *p* = .11, perspective taking, *R^2^* change =.004, *F*(1,199) =.83, *p* = .36, soothing, *R^2^* change <.001, *F*(1,199) =.19, *p* = .67.

**Cognitive Appraisals.** There was no evidence that cultural group moderated the associations between the following cognitive appraisal types and PTSD symptoms; primary control, *R^2^* change =.01, *F*(1,199) = 1.60, *p* = .21, secondary control, *R^2^* change <.01, *F*(1,199) =.35, *p* = .56, non-judgemental, *R^2^* change =.004, *F*(1,199) = 1.06, *p* = .30, psychological inflexibility, *R^2^* change =.005, *F*(1,199) =1.66, *p* = .20, cultural beliefs about adversity, *R^2^* change < .003, *F*(1,199) =.01, *p* = .91, negative self, *R^2^* change =.001, *F*(1,199) =.52, *p* = .47, negative world, *R^2^* change =.003, *F*(1,199) =.92, *p* = .34, external cognitions, *R^2^* change <.01, *F*(1,199) =.84, *p* = .37, or social/cultural self, *R^2^* change = .004, *F*(1,199) =.189, *p* = .17.

**Regression Analyses**

For emotion regulation strategies, the overall model was significant for the Asian American group, *R^2^*=.54, *F*(7,95)=16.02, *p*<.001. The significant unique predictors were general emotion dysregulation, *β*=.34, *t*=2.87, *p*=.005, and trauma-specific rumination, *β*=.48, *t*=4.94, *p<* .001. For the European American group, the overall model was also significant, *R^2^*= .59, *F*(7,96)= 19.98, *p* <.001. The unique predictors were trauma-specific rumination, *β*=.25, *t*=2.46*, p*=.016, and psychological inflexibility, *β*=.44, *t*=3.19, *p*=.002.

For cognitive appraisals, the overall model was significant for the Asian American group, *R^2^*=.51, *F*(6,96)=16.63, *p*<.001. The unique predictors were negative cognitive appraisals of public and communal self, *β*=.48, *t*=4.22, *p*< .001, and negative trauma-related cognitive appraisals, *β*=.26, *t*=2.30, *p*=.02. For the European American group, the overall model was significant, *R^2^*=.68, *F*(6,97)= 34.14, *p* <.001, and the unique predictors were negative cognitive appraisals of public and communal self, *β*=.59, *t*=4.94, *p*<.001, and fatalism appraisals, *β*= .14, *t*=2.06, *p*=.04.
